# Dopamine and memory dedifferentiation in aging

**DOI:** 10.1016/j.neuroimage.2015.03.031

**Published:** 2017-06

**Authors:** Hunar Abdulrahman, Paul C. Fletcher, Edward Bullmore, Alexa M. Morcom

**Affiliations:** a MRC Cognition & Brain Sciences Unit, University of Cambridge, Cambridge, CB2 7EF, UK; b Brain Mapping Unit, Department of Psychiatry, Behavioural and Clinical Neuroscience Institute, University of Cambridge, Robinson Way, Cambridge CB2 0SZ, UK; c Cambridge and Peterborough Foundation trust, Fulbourn Hospital, Cambridge CB21 5EF, UK; d GlaxoSmithKline, ImmunoPsychiatry, Alternative Discovery & Development, Stevenage SG1 2NY, UK; e Centre for Cognitive Ageing and Cognitive Epidemiology, Psychology, University of Edinburgh, Edinburgh EH8 9JZ, UK

**Keywords:** Aging, Dedifferentiation, Episodic memory, Hippocampus, Prefrontal cortex, Dopamine

## Abstract

The dedifferentiation theory of aging proposes that a reduction in the specificity of neural representations causes declines in complex cognition as people get older, and may reflect a reduction in dopaminergic signaling. The present pharmacological fMRI study investigated episodic memory-related dedifferentiation in young and older adults, and its relation to dopaminergic function, using a randomized placebo-controlled double-blind crossover design with the agonist Bromocriptine (1.25 mg) and the antagonist Sulpiride (400 mg). We used multi-voxel pattern analysis to measure memory specificity: the degree to which distributed patterns of activity distinguishing two different task contexts during an encoding phase are reinstated during memory retrieval. As predicted, memory specificity was reduced in older adults in prefrontal cortex and in hippocampus, consistent with an impact of neural dedifferentiation on episodic memory representations. There was also a linear age-dependent dopaminergic modulation of memory specificity in hippocampus reflecting a relative boost to memory specificity on Bromocriptine in older adults whose memory was poorer at baseline, and a relative boost on Sulpiride in older better performers, compared to the young. This differed from generalized effects of both agents on task specificity in the encoding phase. The results demonstrate a link between aging, dopaminergic function and dedifferentiation in the hippocampus.

## Highlights

• We examined memory specificity and its dopaminergic modulation in aging using MVPA.
• Task specificity during encoding was age-invariant.
• Specificity of episodic reinstatement was reduced in older adults in PFC and hippocampus.
• Bromocriptine and Sulpiride modulated these age differences in hippocampus.
• Dopaminergic effects in hippocampus in older adults tracked individual performance.

## Graphical abstract

**Figure:**
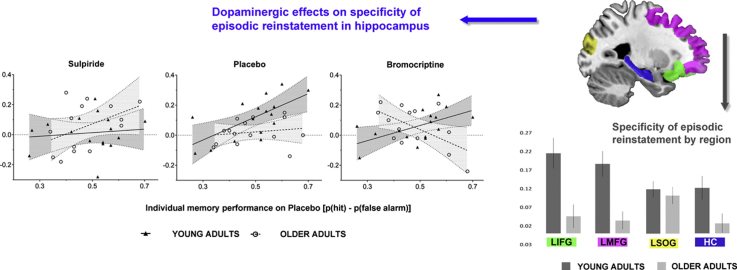

## Introduction

The dedifferentiation theory of cognitive aging proposes that there is a loss of specificity of neural representations as people become older. These pervasive changes are assumed to impact predominantly on the complex cognitive functions which decline the most (Baltes and Lindenberger, 1997, Li et al., 2001). Functional magnetic resonance imaging (fMRI) studies have revealed widespread age-related reductions in the specificity of distributed cortical patterns of activity elicited by different categories of visual stimuli (Carp et al., 2010b, Goh et al., 2010, Park et al., 2004) and different actions (Carp et al., 2011). Preliminary evidence also supports the prediction that dedifferentiation impacts on functions and regions which decline prominently in old age: the visual category-specificity of cortical activity patterns correlates with older adults' fluid processing ability, and varies with working memory load in frontal and parietal cortex (Carp et al., 2010a, Park et al., 2010, Payer et al., 2006). However, little is currently known about the mechanisms of dedifferentiation, nor its impact on episodic memory, one of the cognitive functions most affected by aging. We investigated whether memory representations are less specific in older adults and explored the modulation of memory specificity by dopaminergic drugs.

Normal aging is accompanied by a marked decline in detailed recollection of events, and an increase in false memory (Schacter et al., 1997, Spencer and Raz, 1995). These episodic memory difficulties are typically attributed to declines in the integrity of the prefrontal cortex (PFC) and the hippocampus (e.g., Head et al., 2008, Yonelinas et al., 2007). However, regional age-related changes may be secondary to generalized neural changes such as dedifferentiation. The first aim of the present study was to examine whether the specificity of episodic reinstatement differs according to age. Episodic recollection is thought to involve hippocampal reactivation of stored memory traces which represent events' particular sensory and cognitive properties (Alvarez and Squire, 1994, McClelland et al., 1995). Consistent with this, functional imaging studies show that successful episodic memory retrieval is accompanied by reinstatement of cortical activity associated with the original events (Danker and Anderson, 2010). Studies using multi-voxel pattern analysis (MVPA) have further shown that the specificity of this episodic reinstatement for particular tasks and categories of stimuli varies with strategic memory search and with competition between relevant and irrelevant memories, suggesting that it reflects the specificity of recollection (Kuhl et al., 2011, McDuff et al., 2009). Using MVPA, St-Laurent et al. (2014) recently showed less distinctive cortical reinstatement in older adults for individual items. We examined the specificity of distributed patterns of reinstatement for two different encoding task contexts involving semantic and phonological processing (Johnson et al., 2009, Polyn et al., 2005). We then determined the degree to which distinct task-related activity patterns present during encoding were reinstated during subsequent retrieval, predicting that this measure of memory specificity would be reduced in older relative to younger adults.

According to computational models, age-related dedifferentiation may reflect a reduction in dopamine signaling and neural signal-to-noise in prefrontal cortex (PFC; Li et al., 2001), and potentially elsewhere. Modeling dedifferentiation in this way reproduces disruption of episodic binding functions found in older adults (Li et al., 2005). This is in line with wider evidence of a ‘correlative triad’ between aging, cognition and dopamine function (Bäckman et al., 2006). The second aim of the present study was to extend the findings of our previous report, which examined dopaminergic modulations of brain activity associated with successful episodic encoding across the two encoding tasks (Morcom et al., 2010). The study had a cross-over placebo-controlled design, in which we administered a dopamine agonist (Bromocriptine) and an antagonist (Sulpiride) to manipulate dopamine signaling. Morcom et al. (2010) found age-related differences in dopaminergic effects on activity associated with successful episodic encoding in PFC and hippocampus. This dopaminergic sensitivity was most pronounced in the older adults with poorer memory, consistent with the notion that dopaminergic decline impairs the ability to encode new memories. Specifically, there were reversed subsequent memory (subsequent forgetting) effects within MTL in the older group: i.e., encoding phase activity predicted later forgetting rather than remembering (Morcom et al., 2010). We proposed then that older adults may encode less distinctive memory representations which do not support specific recollection (Morcom et al., 2010, Wagner and Davachi, 2001).

This novel joint analysis of task-specific activity at encoding and its reinstatement at retrieval allowed us directly to test the link between dopamine, aging and dedifferentiation of episodic memory. We predicted that the expected age-related reduction in memory specificity would vary with changes in dopamine signaling. If dopaminergic decline causes dedifferentiation, loss of memory specificity should be dopamine-sensitive. Predictions about the nature of this sensitivity were derived from the results of the successful encoding study (Morcom et al., 2010) and the dopamine aging hypothesis. First, we expected that dopaminergic modulation of memory specificity would track individual differences in memory ability in the older group, and that poorer older performers would show greater dopamine sensitivity, distinguishing them from the young. Second, we predicted that the dopaminergic effect on memory specificity would parallel that previously reported for the univariate memory encoding (subsequent memory) effects. In addition, if the reversed, subsequent forgetting, effects in the older group reflected impaired memory specificity as proposed by Morcom et al. (2010), then Bromocriptine should reduce memory specificity in poorer older performers just as it enhanced subsequent forgetting effects.

## Methods

### Subjects

Sixteen younger (7 female, mean age = 24.9, SD = 4.7 years) and sixteen older adults (9 female, mean age = 66.9, SD = 3.3 years) contributed data. These comprised all subjects from the previous report on the encoding data, as well as 1 young and 3 older subjects who had not provided sufficient data for that event-related analysis, and 1 older participant who contributed data only for the Placebo session. An additional 3 older subjects and 1 young were excluded due to missing Placebo session data (3 with data acquisition or storage issues, 1 withdrew). Therefore, the Placebo condition analyses included 16 young and 16 older subjects, and the drug analyses included samples of 16 and 15. A further older subject was also excluded from analyses of covariance due to an outlier value for the performance covariate, yielding sample sizes of 16 and 14 (see Results: Task specificity and Feature selection, Task specificity and Feature selection). Volunteers were screened on initial telephone contact using a standard questionnaire. The exclusion criteria were a history of any significant psychiatric or physical condition which was likely to affect the brain or cerebral vasculature, current vasoactive or neurotropic medication, and contraindications to the study drugs or to MRI. Each subject also had an electrocardiogram prior to taking part in functional MRI scanning, reviewed by a physician, as well as a structural scan. The groups were matched on years of education (in young, mean = 4.6, SD = 2.6; in old, mean = 4.0, SD = 3.0; t < 1). Estimated verbal IQ using the National Adult Reading Test (Nelson, 1982) was slightly higher in the older group as expected (Backman and Nilsson, 1996); for young, mean = 112, SD = 6.0; for old, mean = 118, SD = 6.5, *t*(34) = 2.96, p = .006; for details see Morcom et al. (2010).

### Experimental design and task

Subjects took part in 3 experimental sessions in which they received Sulpiride 400 mg, Bromocriptine 1.25 mg, or a Placebo orally, in a randomized double-blind crossover design. The scanned episodic memory task commenced after 3 h, and comprised a study (encoding) phase, followed by 2 test (retrieval) phase blocks. To avoid nausea within the double-blind procedure, the study drug was given with 10 mg of the peripheral dopamine antagonist Domperidone (Reddymasu et al., 2007). Subjects were also asked to eat beforehand. For Sulpiride the mean time to maximal plasma concentration is about 3 h, and it has a plasma half-life of around 12 h, and oral bioavailability of about 35%. Plasma prolactin concentration is maximal after about 1 h, then declines slowly (Wiesel et al., 1982, Von Bahr et al., 1991, Caley and Weber, 1995). Bromocriptine's central effects are also long lasting, though somewhat slower to onset than those of Sulpiride, with measurable effects from as early as 1 1/2 h post-dose which maximal after 3 h and persist for some time (Luciana et al., 1998, Müller et al., 1998, Oranje et al., 2004). fMRI data acquisition began at about 3-h post-dose and the sessions were separated by a minimum washout period of a week. Subjects were randomly allocated to each of 6 possible counterbalanced session orders. After exclusions, there were minor imbalances in session ordering between and across age groups. The main analyses are reported with the full N, but we conducted check analyses to rule out possible confounds of session effects: none were found, and effects were if anything more robust once session ordering was balanced. Details of these check analyses are given in the Supplementary material.

**Fig. 1:**
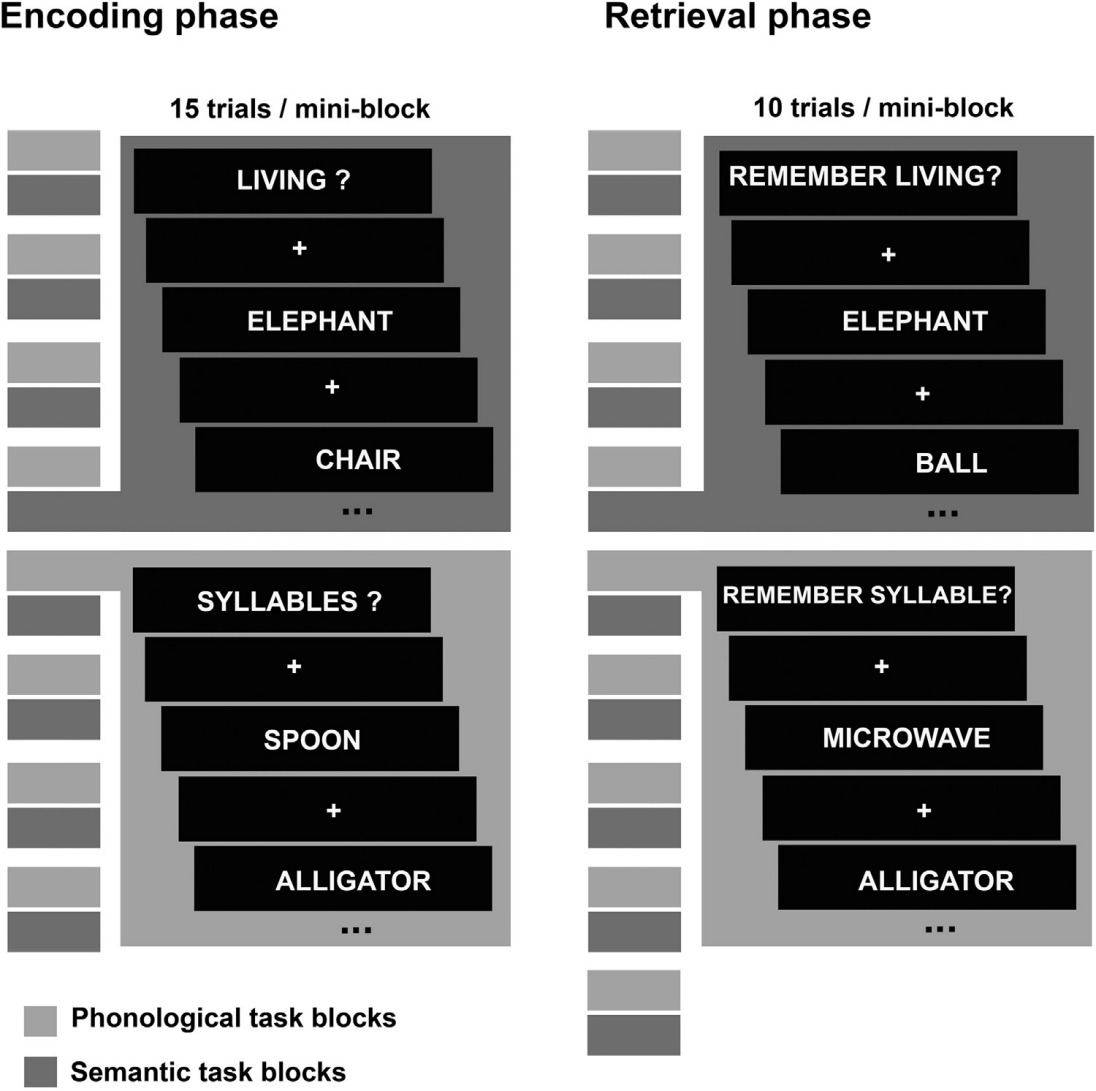
Paradigm design. Illustrates the mini-block structure of the study and test phases of the task. Note that not all mini-blocks are shown. See Experimental design and task for details.

Study and test stimuli were 4–9 letter nouns of 1–3 syllables from the CELEX database (http://www.ru.nl/celex/; for details see Morcom et al., 2010). The paradigm is illustrated in Fig. 1. The study phase consisted of 16 “mini-blocks” of 15 trials each. Subjects performed two different orienting tasks, one involving a semantic and one a phonological judgment. Semantic and phonological mini-blocks alternated and each pair was followed by 21 s fixation. This task ordering was counterbalanced across subjects. Semantic mini-blocks were preceded by the cue “Living?” and subjects judged whether each word referred to a living or a non-living thing. Phonological mini-blocks were preceded by the cue “Syllables?”, and subjects judged whether each word had an even or an odd number of syllables. In both tasks half the items were animate and half inanimate, and of each of these, half had an odd and half an even number of syllables. Items were distributed randomly across mini-blocks. Words were shown center-screen in white uppercase Arial font on a black background. The stimulus onset asynchrony (SOA) at study was 3000 ms, with stimuli on screen for 600 ms followed by fixation.

The test phase consisted of two sessions, each including 18 mini-blocks of 10 trials. The first session immediately followed the study phase (after a brief verbal interaction to prevent rehearsal), followed by the second after an unrelated 6 min task. Subjects were told that in the mini-blocks preceded by the cue, “Remember living”, previously seen items had all been studied in the Living/Non-living task, while in those preceded by “Remember syllable,” they had all been studied in the Syllable task. Two thirds of the items had been studied and a third were new items, distributed randomly across mini-blocks. Subjects judged whether they specifically recollected having studied the word (“Remembered”), whether they thought the word had been studied but it was just familiar (“Know”), or it was unstudied (“New”), using standard “Remember–Know” instructions (Gardiner, 1988). Mini-blocks alternated as at study, with 21 s fixation after each pair. Test phase SOA was 4400 ms, with stimuli on screen for 600 ms followed by fixation.

### MRI data acquisition and preprocessing

Functional scans were acquired using a 3.0T Medspec S300 MRI system, with a gradient-echo echo planar (EPI) pulse sequence (TR = 1200 ms, TE = 27.5 ms, flip angle = 90°). Each EPI volume comprised 23 interleaved 4 mm thick axial slices angled to the intercommissural line, with a 1 mm inter-slice gap (64 × 64 pixels, in-plane resolution 3.125 mm). One encoding timeseries was acquired in the study phase (755 volumes), and two retrieval timeseries in the test phases (825 volumes each). Seven “dummy” volumes were discarded at the start of each run. Outlier scans (with slices of > 5 standard deviations) were replaced with the mean of the 2 neighboring scans.

Initial preprocessing was done in SPM 5 (Wellcome Department of Cognitive Neurology, London, UK; http://www.fil.ion.ucl.ac.uk/spm/software/spm5/). Each timeseries was realigned spatially to the first volume, then normalized using nonlinear basis functions and resampled to 3 × 3 × 3 mm voxels, using an EPI template based on the Montreal Neurological Institute (MNI) reference brain (Cocosco et al., 1997) in the space of Talairach and Tournoux (Ashburner and Friston, 1999, Talairach and Tournoux, 1988). No smoothing was performed. Further preprocessing was carried out in MATLAB 7.6 (www.mathworks.com). Linear trends and frequencies below 1/180 Hz were removed from each timeseries using SPM5's high-pass filter function. The timeseries was then normalized and scaled to a range of (− 1,1) to allow for varying ranges of voxel activity using the Princeton MVPA toolbox (Norman et al., 2006, Detre et al., 2006; http://www.pni.princeton.edu/mvpa/).

### Feature selection

Regions of interest (ROIs) were defined using WFU PickAtlas (http://fmri.wfubmc.edu/). ROIs encompassed lateral PFC (inferior frontal gyrus (IFG) and middle frontal gyrus (MFG)), bilateral hippocampus, and two areas previously shown to be engaged in episodic encoding during the phonological orienting task (bilateral fusiform gyrus (FusG) and left superior occipital gyrus (LSOG; Otten and Rugg, 2001). Prefrontal ROIs were defined for each hemisphere separately (LIFG, RIFG, LMFG & RMFG), as age-related differences in lateralization of memory function in PFC were of potential interest (Morcom, 2003, Cabeza, 2002). Within each ROI, we used the analysis of variance (ANOVA) feature selection utility in the Princeton toolbox to select voxels showing the most significant differences between the two task conditions (semantic and phonological) in each training (encoding phase) dataset. In order to check whether MVPA results varied according to the threshold used for feature selection, we generated 3 separate feature sets for each training dataset and ROI, comprising the 500, 150 and 50 most significant voxels. For each ROI, the best performing feature set in the Placebo condition ridge regression analysis of task specificity (encoding) effects was then used for all subsequent analyses of memory specificity and drug effects, and for the correlation analysis (see Multi-voxel pattern analysis: age-related differences, Task specificity, Multi-voxel pattern analysis: age-related differences, Task specificity, below).

### Multi-voxel pattern analysis using ridge regression

We used multivariate pattern analysis (MVPA) to investigate the specificity of the patterns of neural activity in the semantic and phonological encoding tasks (task specificity), and the specificity with which information encoded using these two tasks was later retrieved (memory specificity). Machine learning algorithms are now widely used to decode neural activity (Polyn et al., 2005, Haynes and Rees, 2006, Kamitani and Tong, 2005). The fidelity with which they can discriminate between two cognitive conditions provides a measure of the distinctiveness of different patterns of neural activation. MVPA measures were computed for each subject and drug condition using the Princeton MVPA toolbox (Norman et al., 2006, Detre et al., 2006; http://www.pni.princeton.edu/mvpa/). We used a penalized ridge regression algorithm because of its sensitivity to intermediate activation values at training and at test, and its ability to compensate for multicollinearity among features (Coutanche et al., 2011, Zhang and Yang, 2003, Poppenk and Norman, 2012). This means that predictions of test set data are continuous rather than binary. To assess the performance of the algorithm for each subject and drug session we calculated the correlation coefficient of its predictions with the labels of the testing set using the inbuilt performance metrics in the Princeton's toolbox, giving test set data values from − 1 to 1 (chance = 0).

The first analyses assessed task specificity, i.e., the distinctiveness of neural patterns during the two orienting tasks (semantic and phonological) within the study phase (encoding). Subjects' encoding timeseries were subdivided into 8 equal subsets, each comprising one mini-block. To account for hemodynamic lag the design was convolved with SPM8's canonical hemodynamic response function (HRF). A ridge regression algorithm was then trained on 7 of these subsets and tested on the 8th in a leave-one-out cross validation procedure with 8 iterations. Before application of the algorithm to the test data, we ran a nested cross-validation procedure on the training data for the Placebo condition to determine the optimum values for the ridge regression penalty parameter which controls the maximum value of the sum of the squares of the voxel weights (Coutanche et al., 2011). The optimum value within the range (0, 0.01, 0.1, 1, 10, 100, 1000, 10,000) did not differ between age groups (median value across ROIs and selected feature sets in both groups = 50; interquartile range = 130, for Kruskal–Wallis tests in each ROI for selected feature sets, p > .05). These individually determined penalty parameters were employed for all subsequent analyses.

Next, we investigated memory specificity in a combined study and test phase (encoding–retrieval) analysis. Memory specificity was defined as the accuracy with which the algorithms trained to discriminate between the encoding tasks were able to predict the retrieval task in each ROI. For this analysis, all 8 pairs of encoding mini-blocks were used as training data, and each retrieval phase's 9 pairs of mini-blocks served as 2 independent test runs. Memory specificity measures were computed for both retrieval phases and the final measure of memory specificity for each subject and drug session was the average performance of the ridge regressor across the two phases. We note that because the encoding and retrieval mini-blocks contained different numbers of trials (15 and 10, see above), this difference could contribute to lower values for memory specificity than for task specificity. However, scan numbers and therefore data points available for the ridge analysis were closely similar between the two phases (37.5 and 36.6). Moreover, an overall difference between levels of task specificity and memory specificity was expected, since they are assumed to reflect very different processes (see Introduction).

### Multi-voxel pattern analysis using correlation distance metric

To check the reproducibility of the ridge regression results and for comparability with prior studies of dedifferentiation in aging, we also measured memory specificity using a correlation distance metric of neural distinctiveness (Carp et al., 2010b, Haxby et al., 2001). To allow for hemodynamic delay, the fixation scans and the first 7 scans of each mini-block were discarded giving 30 scans from each encoding and retrieval mini-block. Voxel values were then averaged across the remaining scans in each semantic and phonological task mini-block for the study and test phases, and across mini-blocks, and Pearson's product moment correlation coefficients computed within and between tasks between the encoding phase and the retrieval phase. Memory specificity was defined as the neural distinctiveness of activity patterns in the two different tasks across the two phases of the episodic memory task. Memory specificity was calculated as the difference between the average correlation within similar tasks (semantic encoding & semantic retrieval and phonological encoding & phonological retrieval) and the average correlation between different tasks (semantic encoding & phonological retrieval and phonological encoding & semantic retrieval).

## Results

### Task performance

Detailed behavioral analyses of both study and test phases are included in the previous report on the encoding data (Morcom et al., 2010). The pattern of findings was unchanged in this larger sample. Performance on the two orienting tasks in the study phase did not differ according to age group or drug condition, and both groups were highly accurate (90% for young, 89% for old). In the test phase, the main index of memory performance was the discrimination index *Pr* for hits and false alarms, collapsed over Remember and Know responses (*P*_hit_ − *P*_false alarm_, Snodgrass and Corwin, 1988). *Pr* did not differ between age groups on Placebo (*t* < 1), but there was a main effect of drug with a linear trend (*F*(1.8, 53.6) = 3.29, p = 0.049; *F*(1,29) = 4.26, p = .048), mainly reflecting a reduction in *Pr* on Sulpiride across both groups (mean = 0.43) relative to Placebo and Bromocriptine (means = 0.47;). As in the previously reported sub-group of subjects, although this effect did not interact with age (*F*(1.8, 53.6) = 1.33), it was driven mainly by a reliable linear effect of drug in the older group taken alone. (Response bias, as indexed with *Br* (*P*_false alarm_ / 1 − (*P*_hit_ − *P*_false alarm_), (Snodgrass and Corwin, 1988)), was also more liberal on Sulpiride (mean = 0.46; for Placebo and Bromocriptine, means = 0.38 and 0.41; values > 0.5 indicate a relatively liberal bias to respond “old”). Valid recollection and familiarity measures were available for a subset of 16 young and 13 older adults; these did not show reliable drug or group effects. In addition, the depth of processing effect (better memory following semantic than phonological encoding; Craik and Lockhart, 1972) did not differ between groups (mean probability of recollection = .53 and .28 in the young respectively, and .50 and .27 in the older group; age effects n.s.) or as a result of the pharmacological manipulation.

### Multi-voxel pattern analysis: age-related differences

#### Task specificity

Encoding phase task specificity in the Placebo condition was assessed using ridge regression, and the results were also used to determine the optimal feature set size for each ROI for the memory specificity and drug analyses (see Methods: Feature selection). Results for all feature sets are given in Inline Supplementary Table S1. Cross-validation showed that the ridge algorithm accurately discriminated between the semantic and phonological orienting tasks in all ROIs and individual subjects (p < 0.01 for all). Average ridge accuracy across ROIs and feature sets was 0.78 in both the young and the older group (individual values ranged in the young group from 0.47 in hippocampus to 0.98 in LIFG; in the older group, from 0.61 in hippocampus to 0.97 in LIFG).

Encoding phase task specificity in the Placebo condition was assessed using ridge regression, and the results were also used to determine the optimal feature set size for each ROI for the memory specificity and drug analyses (see Methods: Feature selection). Results for all feature sets are given in Inline Supplementary Table S1. Cross-validation showed that the ridge algorithm accurately discriminated between the semantic and phonological orienting tasks in all ROIs and individual subjects (p < 0.01 for all). Average ridge accuracy across ROIs and feature sets was 0.78 in both the young and the older group (individual values ranged in the young group from 0.47 in hippocampus to 0.98 in LIFG; in the older group, from 0.61 in hippocampus to 0.97 in LIFG).

Inline Supplementary Table S1

**Table S1:** Task specificity (study phase) under Placebo (ridge regression). The mean (SD) accuracy of discrimination between semantic and phonological task blocks in the study (encoding) phase of the task is listed (see Methods for details of analyses and Results for statistical analysis).

ROI/# voxels	Younger group			Older group		
	50	150	500	50	150	500
LIFG (500)	0.82 (0.07)	0.83 (0.07)	0.84 (0.07)	0.80 (0.07)	0.81 (0.07)	0.82 (0.07)
RIFG (500)	0.80 (0.07)	0.81 (0.08)	0.80 (0.09)	0.76 (0.06)	0.77 (0.07)	0.77 (0.08)
LMFG (500)	0.80(0.07)	0.82 (0.06)	0.83 (0.06)	0.80 (0.06)	0.82 (0.05)	0.83 (0.05)
RMFG (500)	0.80 (0.06)	0.83 (0.06)	0.84 (0.06)	0.80 (0.07)	0.81 (0.07)	0.82 (0.07)
HC (50)	0.69 (0.04)	0.69 (0.05)	0.63 (0.09)	0.72 (0.07)	0.72 (0.07)	0.70 (0.09)
LSOG (150)	0.73 (0.07)	0.73 (0.08)	0.71 (0.08)	0.76 (0.08)	0.77 (0.09)	0.77 (0.09)
FusG (150)	0.76 (0.06)	0.78 (0.06)	0.78 (0.07)	0.76 (0.06)	0.78 (0.06)	0.77 (0.07)

Inline Supplementary Table S1

The feature sets selected for each ROI were those with the maximum ridge performance on Placebo which avoided any confounds of training set performance with age. Ridge accuracy was better for larger feature sets in PFC, and this did not differ according to age. Therefore the 500 voxel feature sets were selected for memory specificity and drug analyses for these ROIs. In HC, task specificity did not differ according to age and was greatest for the smaller feature sets, so these were used for further analyses. In LSOG, the intermediate feature sets of 150 voxels were selected to balance for the slight (but non-significant) increase in task specificity with # voxels in the older group, and decrease in the young. In FusG, the 150 voxel feature set was selected, in which task specificity was maximal and equivalent across age groups.

We also tested for associations between encoding phase task specificity and individual differences in performance in the selected feature sets using ANCOVA with covariates of mean-corrected *Pr* (see Results: Task performance for definition) and the interaction of *Pr* x group (one older subject was excluded from these analyses due to an outlier *Pr* value, > 2.5 SD from the mean). These used *Pr* on Placebo as the covariate. These showed no associations in IFG or MFG (max *F* = 1.11). In posterior ROIs, behavioral associations were not reliable. Marginally significant main effects of *Pr* in HC and FusG (p = .089; p = .063) reflected trends for task specificity to be greater in better performers across both age groups; such trends could not complicate the interpretation of any age-related differences in memory specificity or in dopaminergic drug effects.

#### Memory specificity

**Fig. 2:**
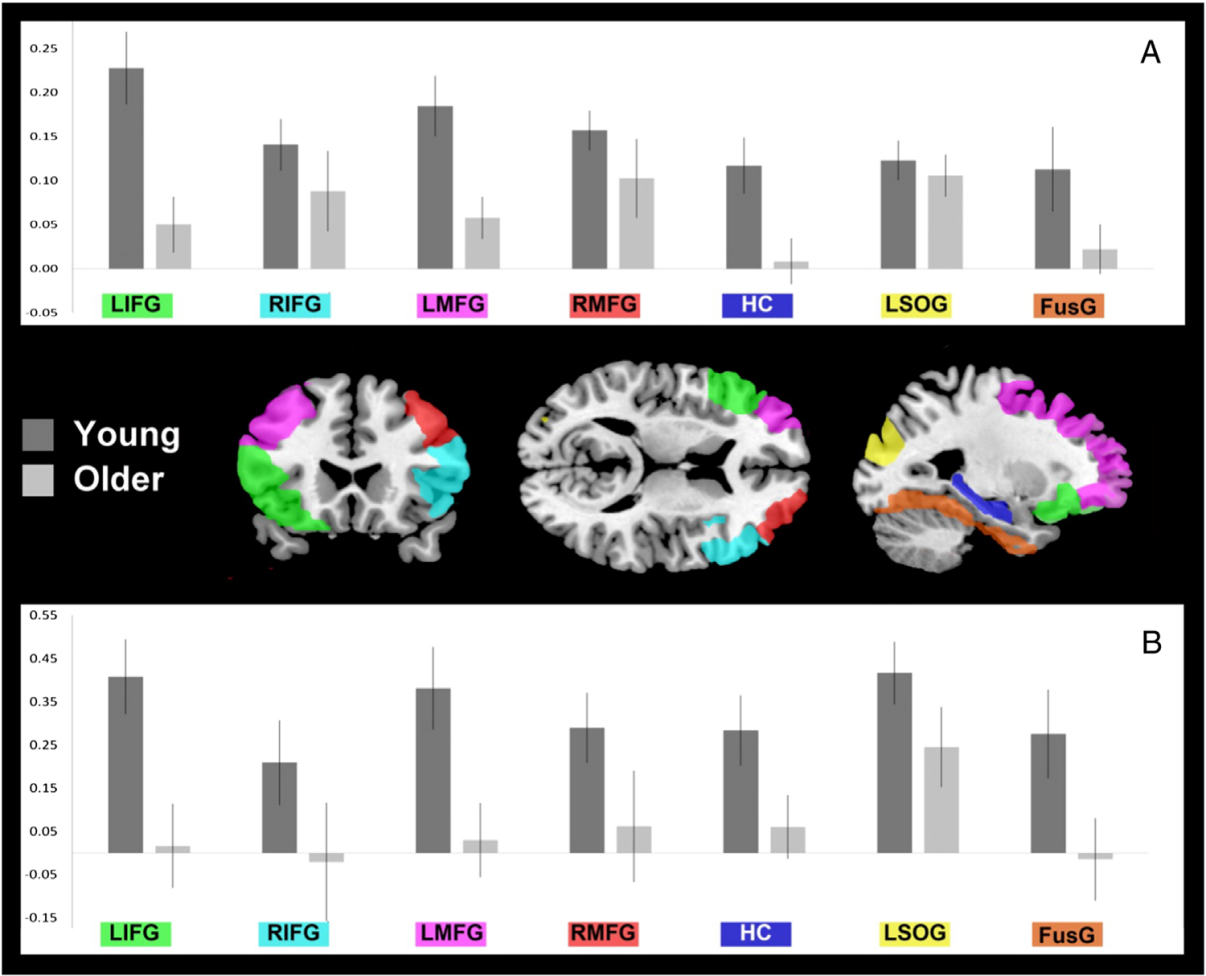
Age-related differences in memory specificity (Placebo session). ROIs are overlaid on the T1 MNI template from MRIcron (http://www.mccauslandcenter.sc.edu/mricro/mricron/; sections at *x* = 30, *y* = 18, *z* = 12). A. Plots show accuracy of the ridge regression for predicting the task at retrieval when trained to discriminate the tasks at encoding (chance = 0). Mean accuracy across feature set sizes is shown for each age group. B. Plots show the mean correlation distance metric between encoding and retrieval (within-task correlation–between-task correlation). Error bars represent the within-group standard error of the mean. See Methods for details of measures and Results for details of analyses.

**Fig. 3:**
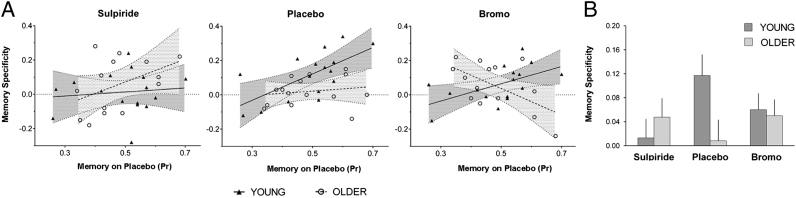
Dopaminergic modulation of memory specificity in hippocampus assessed using ridge regression. A. Scatter plots show the relation between memory specificity (y-axis) and baseline individual memory performance — (x-axis) in young and older age groups in the 3 drug conditions. Baseline individual memory performance is indexed by *Pr* on Placebo. Best fit regression lines of memory specificity to Baseline *Pr* within each age group and drug condition are also shown (note that although raw *Pr* values are given here, ANCOVA analyses used within-group mean corrected *Pr* values; see Results). B. The bar graph shows mean memory specificity for each age group and drug condition. Error bars represent the within-group standard errors.

The results of the encoding–retrieval memory specificity analysis for the Placebo condition are illustrated in Fig. 2, Fig. 3. For each ROI, ridge regression MVPA measures of memory specificity for the selected feature sets were subjected to ANOVA with the factor of age group. Further analyses with the additional factor of hemisphere tested for lateralization differences where group differences were apparent in one ROI. We then tested for brain–behavior associations using ANCOVA with the additional covariates of *Pr* (on Placebo) and *Pr* x group (see Task specificity and Feature selection, Task specificity and Feature selection). Where covariate effects were present, we checked that these remained significant when individual age was also included in the model, to rule out potential confounds between performance- and age-related effects within groups (Hofer and Sliwinski, 2001). Except where noted, this was the case. Following ridge analyses, we conducted replication analyses using the correlation distance metric to assess consistency of results across MVPA metrics. These are reported where there were positive findings from the ridge analysis. In summary, consistent age-related differences in memory specificity were found in left PFC (LIFG and LMFG) and in hippocampus.

##### Prefrontal cortex

In LIFG, memory specificity assessed with ridge regression was reduced in the older group relative to the young (*F*(1,30) = 9.09, p = 0.005; for replication with correlation distance metric (*F*(1,30) = 15.80; p *<* 0.001). In the older group, memory specificity was not significantly greater than chance. In RIFG, group differences were not reliable (*F* < 1), but effects did not vary significantly by hemisphere (for interaction with group, *F*(1,30) = 1.48, p = 0.233). Direct comparison with encoding phase neural specificity measures also confirmed that the age-related reduction in memory specificity was significantly greater than (non-significant) group differences in task specificity at encoding (for group x task phase, *F*(1,27) = 5.12, p = 0.032). ANCOVA showed no brain–behavior associations in LIFG. In RIFG, there was an association between memory specificity and memory performance across groups (for ridge, *F*(1,27) = 4.39, p = 0.049; for correlation, *F*(1,27) = 6.65, p = 0.017), although significance was reduced with age in the model, for ridge, *F*(1,26) = 1.92, n.s.; for correlation, *F*(1,26) = 5.86, p = 0.023). Analysis across task specificity and memory specificity ridge regression measures showed that this association with performance was common to both, as reflected in a significant main effect of *Pr* (*F*(1,27) = 5.02, p = 0.017; for task x *Pr*, *F*(1,27) = 1.73, n.s.).

Ridge analysis for left middle frontal gyrus (LMFG), as in LIFG, revealed a group difference in memory specificity favoring the young (*F*(1,30) = 7.08, p = 0.012; for replication analysis with correlation, *F*(1,30) = 8.74, p = 0.006), with ridge accuracy again at chance in the older group. As in LIFG, direct comparison confirmed that the group difference was driven by memory specificity relative to encoding phase task-specificity (for task phase main effect, *F*(1,30) = 7.94, p = 0.008). In RMFG, as in RIFG, group differences were not significant (*F*(1,30) = 1.2, n.s.), but laterality analysis did not show reliable age-related differences by hemisphere. Brain–behavior analysis in MFG did not reveal any significant findings.

Because the correlation measure of neural distinctiveness is a function of correlations both within and between tasks, age differences in memory specificity could be driven by effects on within-task correlations, between-task correlations, or both (see Carp et al., 2010b). *Post hoc* tests in PFC showed that both within-task and between-task correlation effects contributed to the group differences in LIFG (main effect of group for within- *F*(1,30) = 12.8 p = 0.001; for between-, *F*(1,30) = 13.1, p = 0.001) and in LMFG (for within-, *F*(1,30) = 9.3 p = 0.005; for between-, *F*(1,30) = 15.2, p < 0.001).

##### Hippocampus

In HC, ridge analysis showed reduced memory specificity in the older group (*F*(1,30) = 6.50, p = 0.016). There was also a positive association between memory specificity and memory performance (for *Pr*, *F*(1,27) = 8.77, p = 0.006) and a marginal age-related difference in this association (for group x *Pr*, *F*(1,27) = 3.12, p = 0.089). The presence of robust group differences in the association between memory specificity and memory performance was confirmed by a direct comparison between task specificity at encoding (for which brain–behavior associations were non-significant; see last section) and memory specificity. This revealed a significant interaction between task phase, group and *Pr* (*F*(1,27) = 4.59, p = 0.041). Correlation analysis replicated the interaction of group with memory performance (for group x *Pr*, *F*(1,26) = 6.17, p = 0.019). In the young only, memory specificity was robust for both measures (*F*(1,14) = 10.93, p = 0.005 for ridge; *F*(1,15) = 5.75, for correlation, p = 0.030) and was positively associated with performance (*F*(1,14) = 10.86, p = 0.005 for ridge; *F*(1,14) = 9.71, p = 0.008 for correlation).

##### Posterior cortex

There were no reliable age-related differences in memory specificity in the posterior ROIs on Placebo. In FusG, ridge analysis did not show reliable age-related differences in memory specificity (*F*(1,30) = 2.57, p = 0.119), nor significant brain–behavior associations (for *Pr*, *F*(1,27) = 4.01, p = 0.056; for group x *Pr*, *F*(1,27) = 3.82, p = 0.062). As in RIFG, analysis across neural specificity measures for both task phases showed a positive overall relation with individual performance across age groups (for *Pr* main effect, *F*(1,27) = 6.23, p = 0.019; for interaction with task phase, *F* = 1.23).

In LSOG, memory specificity was age-invariant (for group, *F* < 1) and robust across age groups (for intercept across age groups *F*(1,30) = 38.73, p < 0.001 for ridge, *F*(1,30) = 28.43, p < 0.001 for correlation). It did not vary with individual memory performance (*F* < 1 for *Pr* effects).

### Multi-voxel pattern analysis: dopaminergic drug effects

#### Encoding phase: task specificity

There was a pronounced age-invariant dopaminergic modulation of the ridge measure of task specificity in all ROIs (see Table 1; for drug, min *F* = 22.68, max p < 0.001; for group x drug, max *F* = 1.11, min p = 0.335). In both age groups, task specificity was increased by both Sulpiride and Bromocriptine relative to Placebo (for pairwise contrasts, all p < 0.001). However, no age-dependent dopaminergic effects were observed. The drugs did not modulate brain–behavior relations.

**Table 1:** Drug effects on encoding phase task specificity (ridge regression). Means (SDs) are given for analyses of the selected feature sets in the Sulpiride, Placebo and Bromocriptine conditions (see Table 1).

ROI (# voxels)/drug session	Younger group			Older group		
	Sulpiride	Placebo	Bromocriptine	Sulpiride	Placebo	Bromocriptine
LIFG (500)	0.93 (0.03)	0.84 (0.07)	0.92 (0.05)	0.92 (0.05)	0.82 (0.07)	0.90 (0.06)
RIFG (500)	0.89 (0.08)	0.80 (0.09)	0.89 (0.09)	0.90 (0.06)	0.77 (0.08)	0.90 (0.06)
LMFG (500)	0.92 (0.06)	0.83 (0.06)	0.92 (0.05)	0.92 (0.04)	0.83 (0.05)	0.91 (0.06)
RMFG (500)	0.92 (0.06)	0.84 (0.06)	0.92 (0.05)	0.92 (0.07)	0.82 (0.07)	0.92 (0.04)
HC (50)	0.84 (0.07)	0.69 (0.04)	0.83 (0.07)	0.83 (0.09)	0.72 (0.07)	0.85 (0.08)
LSOG (150)	0.88 (0.05)	0.73 (0.08)	0.88 (0.08)	0.88 (0.06)	0.77 (0.09)	0.87 (0.09)
FusG (150)	0.86 (0.06)	0.78 (0.06)	0.87 (0.06)	0.87 (0.09)	0.78 (0.06)	0.88 (0.07)

#### Memory specificity

In hippocampus there was a dopaminergic modulation of the age-related differences in memory specificity which varied with individual differences in memory performance, illustrated in Fig. 3. Memory specificity did not show reliable dopaminergic effects in PFC or posterior ROIs; details of these analyses are not reported (summary data for all ROIs are given in Table S2, Table S3).

Inline Supplementary Table S2

**Table S2:** Drug effects on memory specificity (ridge regression). Means (SDs) are given for analyses of the selected feature sets in the Sulpiride, Placebo and Bromocriptine conditions (see Table 1 for details).

ROI (# voxels)/drug session	Younger group			Older group		
	Sulpiride	Placebo	Bromocriptine	Sulpiride	Placebo	Bromocriptine
LIFG (500)	0.14 (0.11)	0.23 (0.18)	0.16 (0.14)	0.11 (0.14)	0.05 (0.15)	0.13 (0.13)
RIFG (500)	0.08 (0.14)	0.14 (0.12)	0.07 (0.12)	0.09 (0.15)	0.09 (0.18)	0.03 (0.14)
LMFG (500)	0.14 (0.08)	0.18 (0.14)	0.16 (0.13)	0.08 (0.14)	0.06 (0.13)	0.08 (0.11)
RMFG (500)	0.08(0.09)	0.16 (0.12)	0.07 (0.17)	0.12 (0.11)	0.10 (0.16)	0.11 (0.14)
HC (50)	0.01 (0.12)	0.12 (0.14)	0.06 (0.10)	0.05 (0.15)	0.01 (0.10)	0.05 (0.13)
LSOG (150)	0.07 (0.11)	0.12 (0.11)	0.15 (0.15)	0.08 (0.15)	0.11 (0.09)	0.06 (0.18)
FusG (150)	0.08 (0.15)	0.11 (0.18)	0.02 (0.12)	0.05 (0.15)	0.02 (0.14)	0.05 (0.17)

Inline Supplementary Table S2

Inline Supplementary Table S3

**Table S3:** Drug effects on memory specificity (correlation distance metric). Means (SDs) are given for the selected feature sets for the difference: within-task correlation — between task correlation. Both correlation measures are among semantic and phonological task blocks across the study (encoding) phase and the test (retrieval) phase of the task (see Methods for details of analyses and Results for statistical analysis).

ROI (# voxels)/drug session	Younger group			Older group		
	Sulpiride	Placebo	Bromocriptine	Sulpiride	Placebo	Bromocriptine
LIFG	0.22 (0.38)	0.41 (0.27)	0.40 (0.35)	0.04 (0.45)	0.02 (0.28)	0.08 (0.45)
RIFG	0.09 (0.38)	0.21 (0.28)	0.09 (0.33)	0.14 (0.42)	− 0.02 (0.43)	0.00 (0.38)
LMFG	0.21 (0.37)	0.38 (0.34)	0.41 (0.44)	0.03 (0.59)	0.03 (0.34)	0.09 (0.48)
RMFG	0.08 (0.35)	0.29 (0.27)	0.15 (0.53)	0.14 (0.50)	0.06 (0.44)	0.12 (0.41)
HC	0.10 (0.36)	0.28 (0.47)	0.15 (0.48)	0.11 (0.53)	0.06 (0.37)	− 0.07 (0.64)
LSOG	0.16 (0.33)	0.42 (0.30)	0.20 (0.66)	0.18 (0.52)	0.25 (0.37)	0.11 (0.66)
FusG	0.15 (0.43)	0.28 (0.42)	0.11 (0.41)	0.09 (0.46)	− 0.01 (0.41)	− 0.02 (0.52)

Inline Supplementary Table S3

Ridge analysis in hippocampus revealed that young and old groups differed in drug effects on the association of memory specificity with *Pr* (see Fig. 3; for group x drug x *Pr*, *F*(1.7,43.3) = 6.85, p = 0.004; for drug x *Pr*, *F*(1.7,43.3) = 4.49, p = 0.022; for group x drug, *F*(1.8,51.7) = 2.54, p = 0.095). The correlation analysis replicated the interaction of group with drug and *Pr* (*F*(2.0,50.9) = 4.66, p = 0.014). Critically, as for the baseline age-related effects, direct comparison between the ridge neural specificity measures in the two task phases showed that the age-dependent modulation of memory specificity was distinct from the age-invariant modulation of encoding phase task specificity described above for HC and in the other ROIs (for task phase x drug x group x *Pr*, *F*(1.6,42.8) = 5.66, p = 0.010).

*Post hoc* tests in the young revealed dopaminergic modulation of memory specificity regardless of performance (for drug, *F*(1.6,22.2) = 4.42, p = 0.031), with a quadratic trend reflecting reduction in memory specificity on both Sulpiride and Bromocriptine relative to Placebo (*F*(1,14) = 8.02, p = 0.013). This group also showed a dopamine-insensitive positive relation between memory specificity and memory performance (for *Pr* main effect, *F*(1,14) = 8.16, p = 0.013; for drug x *Pr*, *F* = 2.32, p = 0.130). In the older group, drug effects varied according to individual differences in memory performance (for drug x *Pr*, *F*(1.7,20.7) = 6.96, p = 0.006 for ridge and *F*(1.8,21.6) = 6.90, p = 0.006 for replication with correlation metric), with a clear linear trend from the Sulpiride through Placebo to the Bromocriptine condition (*F*(1,12) = 10.62, p = 0.007 for ridge, *F*(1,12) = 15.05, p = 0.002 for correlation).

Within the older group, this memory specificity effect also differed reliably from any drug effects on encoding phase task specificity (for task phase x drug x *Pr*, *F*(1.8,22.0) = 5.01, p = 0.018). The only discrepancy between the ridge and correlation indices of memory specificity was that although both showed a strong linear trend, the ridge measure suggested a predominant Bromocriptine effect (see Fig. 3; for pairwise comparison with Placebo for drug x *Pr*, *F*(1,12) = 12.63, p = 0.004 for ridge; *F*(1,12) = 1.53, p = 0.240 for correlation), while the correlation metric suggested a predominant Sulpiride effect (*F*(1,12) = 6.62, p = 0.024 for correlation; *F* < 1 for ridge). While on Placebo memory specificity did not vary with performance in the older group (*F* < 1 for both measures), Bromocriptine induced a more negative association between memory specificity and performance, with memory specificity increasing in poorer performers and decreasing in better performers within the older group (for *Pr* effect on Bromocriptine *F*(1,12) = 7.56, p = 0.018 for ridge; *F*(1,12) = 1.24, p = 0.288 for correlation). Sulpiride had the opposite effect, inducting a more positive association of memory specificity and *Pr* (*F*(1,12) = 3.27, p = 0.096 for ridge, *F*(1,12) = 11.01, p = 0.006 for correlation).

*Post hoc* tests were also conducted with individual linear drug effects on memory specificity as the dependent measure (on Bromocriptine–Sulpiride). These confirmed reliable interactions of age group and *Pr* (for ridge, *F*(1,26) = 11.77, p = .022; for correlation, *F*(1,26) = 6.55, p = .017). Analyses of the relations between linear performance effects (*Pr* on Bromocriptine–Sulpiride) and linear drug effects did not reveal any significant effects (*F* < 1 for all).

## Discussion

Our results show that contextual reinstatement during episodic memory retrieval is less specific in older adults, as predicted by the dedifferentiation account of cognitive aging (Carp et al., 2010b, Li et al., 2001, Park et al., 2004). The data support the proposal that age-related dedifferentiation impacts on episodic memory and impairs memory specificity (Li et al., 2005, St-Laurent et al., 2014). In both young and older age groups, highly specific distributed patterns of neural activity distinguished the processing of semantic and phonological task contexts during the encoding phase, but reinstatement of these task-related patterns at retrieval – memory specificity – was reduced in the older adults in PFC and hippocampus. This reduction in the distinctiveness of retrieved representations was not accounted for by age-related differences in the specificity with which the original task contexts were represented. Task specificity and memory specificity also showed dissociable dopaminergic sensitivity with age-invariant and age-dependent effects, respectively. In hippocampus, memory specificity varied linearly with dopamine stimulation in the older group and this modulation tracked individual differences in memory performance. The dopaminergic effect in hippocampus was distinct from a generalized age-invariant increase in task specificity on both Sulpiride and Bromocriptine. Our data support the notion that dopaminergic function in old age impacts hippocampal memory processes (Chowdhury et al., 2012, Kaasinen et al., 2000, Morcom et al., 2010, Stemmelin et al., 2000, Wilson et al., 2006).

Findings in hippocampus under Placebo were as predicted. The robust reinstatement of task-specific activity during episodic retrieval in the young group is consistent with recent reports that elements of specific memory traces within the hippocampus are reactivated during recollection (Chadwick et al., 2011, Staresina et al., 2012; but see Ritchey et al., 2013), although at the current spatial resolution activity in adjacent cortical regions cannot be excluded. Hippocampal reinstatement was not detectable in the older adults, even though distinctiveness of the original two task contexts was, if anything, slightly greater in this group. This is the first report of an age-related reduction in memory specificity in hippocampus and the first to use trial-unique stimuli, converging with recent findings in cortical regions for reinstatement at the level of individual items (St-Laurent et al., 2014). Models of hippocampal function specify that it is critical for the pattern separation of distinct memory traces for highly similar events and their later reinstatement by pattern completion (Marr, 1982, O'Reilly and McClelland, 1994, Treves and Rolls, 1994), functions which appear to be compromised in aging (Wilson et al., 2006, Yassa et al., 2011).

It is important to note that the group difference in neural memory specificity did not reflect a simple absence of recollection in the older adults: recollective experience was just as likely in this group, and received the same boost from semantic as opposed to phonological processing. Instead, the findings indicate a reduction in the distinctiveness of reinstatement assumed to support contextual recollection (Danker and Anderson, 2010, St-Laurent et al., 2014). Recovery of episodic detail is typically impoverished in older adults even when subjective recollection occurs (e.g. Levine et al., 2002). Our findings indicate that the decline in recollection of episodic detail in old age (Schacter et al., 1997, Spencer and Raz, 1995) is accompanied by a reduction in the distinctiveness of contextual representations. The data suggest an age-related reduction in the specificity of hippocampal encoding, storage and/or retrieval of these representations which impacts on their later reinstatement during recollection.

Age-related reductions in memory specificity in left dorsolateral and ventrolateral PFC were prominent while memory specificity was age-invariant in LSOG. However, the data do not necessarily suggest selective anterior changes as predicted by the frontal aging hypothesis (West, 1996): although group differences were not clear cut in fusiform gyrus, memory specificity in that region was numerically greater in the young and non-significant in the older adults, consistent with other studies (Carp et al., 2010a, Carp et al., 2010b, Carp et al., 2011, Goh et al., 2010, Park et al., 2010, Park et al., 2012, St-Laurent et al., 2014). Critically, as in hippocampus, the group differences in cortical memory specificity were task-dependent: representations of task context in the encoding phase were well-differentiated in both age groups, unlike contextual reinstatement. It is fundamental to the neural dedifferentiation hypothesis that less differentiated representations be able to explain the marked age-related declines in higher-order functions, notably fluid intelligence, processing speed and – as examined in the present study – episodic memory (Li et al., 2001). Our results support this proposal, as do recent demonstrations of associations between neural category-specificity in older adults and fluid processing (Park et al., 2010), working memory load (Carp et al., 2010a), and episodic memory rather than perception (St-Laurent et al., 2014). In terms of brain–behavior relations, the present study also shows for the first time an association between an index of representation specificity and task performance which is age-dependent. This is consistent with the assumption of the dedifferentiation account that declines in specificity accounts of age-related cognitive change.

The results of our psychopharmacological manipulation provide some support for the theory that a decline in dopamine transmission underpins age-related dedifferentiation (Li et al., 2001). In hippocampus, Sulpiride induced greater memory specificity in older adults whose memory was better at baseline (on Placebo) relative to those whose memory was poorer. The resulting brain–behavior association for the group as a whole on Sulpiride resembled that in the young on Placebo. Conversely, Bromocriptine induced a negative association of memory specificity and memory performance in the older group, boosting memory specificity in poorer relative to better performers (see Fig. 3). This partially supports our first prediction, and our prior findings (Morcom et al., 2010), indicating an association between dopaminergic-sensitivity of memory processing and individual memory ability in older adults only. However this association did not involve just a greater sensitivity in poorer performers, but a varying pattern of response according to baseline level of performance. While consistent with the dopamine hypothesis of aging, this does not fit the simple view that dopaminergic decline both reduces memory performance and increases dopamine sensitivity via a single mechanism. This result is considered in more detail below. The finding of an age- and individual performance-related dopaminergic modulation of hippocampal memory specificity, and the findings of Morcom et al. (2010), are also in line with recent behavioral genetics data which implicate individual differences in dopamine receptor and transporter genotypes in individual differences in episodic memory in later life (Li et al., 2013, Papenberg et al., 2013, Papenberg et al., 2014).

As noted in the Introduction, we previously found that encoding phase activity in the older group in MTL predicted later forgetting rather than remembering, and proposed that older adults may encode less distinctive memory representations which may not support specific recollection (Morcom et al., 2010). This is consistent with the current findings under Placebo. However, the dopaminergic effects in the present study suggest a need for modification of our previous account of the subsequent forgetting effects. This predicts that an intervention which enhances the subsequent forgetting effects would also tend to reduce memory specificity. However, Bromocriptine increased memory specificity in older adults with poorer memory at the same time as enhancing subsequent forgetting effects (see Fig. 3). The latter effects may instead reflect a form of “partial compensation”, which may improve subsequent memory specificity when it is engaged but may be engaged only when there has been some underlying loss of memory function (Daselaar and Cabeza, 2005, de Chastelaine et al., 2011, Morcom and Johnson, in press). This would be in keeping with the linear increase in memory performance in the older group with the increase in dopamine signaling, alongside the subsequent forgetting effects in the older group, i.e., association of activity in this region with unsuccessful encoding (although the behavioral effect did not vary reliably with individual differences in performance).

The dopaminergic modulation of distributed task-specific activity in the encoding phase was unexpected, with age-invariant increases under both Sulpiride and Bromocriptine. There were no accompanying behavioral effects on the phonological and semantic decisions, although the age-invariant Sulpiride effect on decision criterion in the memory task may reflect neuromodulatory mechanisms also affecting processing during one or both of the two orienting tasks. The task specificity measure was included as a baseline for the memory specificity measure, and likely reflected a range of linguistic, mnemonic and executive processes engaged in the two tasks. In pharmacological neuroimaging, nonspecific effects of drugs such as modulations of cerebral blood flow are a potential concern (Honey and Bullmore, 2004). These seem unlikely to account for highly process-specific effects such as those on memory specificity, but might contribute to the widespread effects on task specificity. Whatever the nature of the latter effect, the critical point for interpretation of the episodic memory findings is that the age-dependent dopaminergic modulation of memory specificity in hippocampus differed clearly from the age-invariant effects on task specificity. The performance-related drug effects in the older group only are consistent with the literature suggesting age-related changes in dopaminergic neuromodulation and reveal a greater general sensitivity to perturbations in dopamine signaling than in the young.

Our current and earlier investigations converge to support the possibility that age-related memory impairment is associated with an imbalance in hippocampal dopaminergic regulation. Older adults were more sensitive to dopaminergic perturbation than the young: D2-like blockade was associated with improved memory function (greater hippocampal memory specificity) in better older performers and D2-like stimulation with improved function in poorer performers. A hippocampal locus of this effect is consistent with associations of aging and age-related memory decline with loss of dopamine neurons and D2-like receptors in this region (Kaasinen et al., 2000) (Stemmelin et al., 2000). Dopamine regulates hippocampal function by modulation of its cortical inputs, directly via CA1 (Otmakhova and Lisman, 1998) and indirectly via entorhinal cortex (Pralong and Jones, 1993, Caruana, 2006). Thus the direction of effects may depend on cortical inputs as well as baseline function (Fujishiro et al., 2005, Umegaki et al., 2001). Behavioral and neuroimaging investigations in humans have found that D2-like modulation can enhance or impair cognitive function according to baseline function (e.g., Mehta et al., 2005, Mehta et al., 2008, Reeves et al., 2010), consistent with the literature on inverted U functions in PFC (see Cools and D'Esposito, 2011) and their alteration in aging (Mattay et al., 2006), as well as with the present data.

Given the systemic dopaminergic manipulation, however, it is also possible that upstream effects – for example in striatum – can explain the MTL responses (Honey and Bullmore, 2004, Morcom et al., 2010). We found no evidence that the age-related differences in memory specificity in PFC were mediated by changes in dopaminergic transmission (Braver et al., 2001, Li et al., 2001). However, this null finding requires cautious interpretation. Future studies should investigate the possibility that the critical age changes mediating memory dedifferentiation in lateral PFC involve D1-like receptors which are numerous in this region (Bäckman et al., 2011). Whether or not cortical dopaminergic decline impacts on episodic memory, our findings in MTL are at least a marker of dopaminergic dysregulation, and hint that it may be possible to improve this regulation by adjusting dopamine signaling. Future studies are needed to establish the behavioral as well as the neural impact of such adjustments.

## References

[bib0005] Alvarez P., Squire L.R. (1994). Memory consolidation and the medial temporal-lobe — a simple network model. Proc. Natl. Acad. Sci. U. S. A..

[bib0010] Ashburner J., Friston K.J. (1999). Nonlinear spatial normalization using basis functions. Hum. Brain Mapp..

[bib0015] Backman L., Nilsson L.-G. (1996). Semantic memory functioning across the adult life span. Eur. Psychol..

[bib0035] Bäckman L., Nyberg L., Lindenberger U., Li S.C., Farde L. (2006). The correlative triad among aging, dopamine, and cognition: current status and future prospects. Neurosci. Biobehav. Rev..

[bib0030] Bäckman L., Karlsson S., Fischer H., Karlsson P., Brehmer Y., Rieckmann A., Macdonald S.W., Farde L., Nyberg L. (2011). "Dopamine D 1 receptors and age differences in brain activation during working memory.". Neurobiol. Aging.

[bib0020] Baltes P.B., Lindenberger U. (1997). Emergence of a powerful connection between sensory and cognitive functions across the adult life span: a new window to the study of cognitive aging?. Psychol. Aging.

[bib0025] Braver T.S., Barch D.M., Keys B.A., Carter C.S., Cohen J.D., Kaye J.A., Janowsky J.S., Taylor S.F., Yesavage J.A., Mumenthaler M.S., Jagust W.J., Reed B.R. (2001). Context processing in older adults: evidence for a theory relating cognitive control to neurobiology in healthy aging. J. Exp. Psychol. Gen..

[bib9000] Cabeza R. (2002). Hemispheric asymmetry reduction in older adults: The HAROLD model. Psychol. Aging.

[bib0040] Caley C.F., Weber S.S. (1995). Sulpiride: an antipsychotic with selective dopaminergic antagonist properties. Ann. Pharmacother..

[bib0045] Carp J., Gmeindl L., Reuter-Lorenz P.A. (2010). Age differences in the neural representation of working memory revealed by multi-voxel pattern analysis. Front. Hum. Neurosci..

[bib9005] Caruana Douglas A. (2006). Dopamine has bidirectional effects on synaptic responses to cortical inputs in layer II of the lateral entorhinal cortex. J. Neurophysiol..

[bib0055] Carp J., Park J., Polk T.A., Park D.C. (2010). Age differences in neural distinctiveness revealed by multi-voxel pattern analysis. NeuroImage.

[bib0050] Carp J., Park J., Hebrank A., Park D.C., Polk T.A. (2011). Age-related neural dedifferentiation in the motor system. PLoS ONE.

[bib0060] Chadwick M.J., Hassabis D., Maguire E.A. (2011). Decoding overlapping memories in the medial temporal lobes using high-resolution fMRI. Learn. Mem..

[bib0065] Chowdhury R., Guitart-Masip M., Bunzeck N., Dolan R.J., Düzel E. (2012). Dopamine modulates episodic memory persistence in old age. J. Neurosci..

[bib0070] Cocosco C.A., Kollokian V., Kwan R.K.S., Evans A.C. (1997). Brainweb: online interface to a 3D MRI simulated brain database. NeuroImage.

[bib0075] Cools R., D'Esposito M. (2011). Inverted-U-shaped dopamine actions on human working memory and cognitive control. Biol. Psychiatry.

[bib9010] Coutanche Marc N., Thompson-Schill Sharon L., Schultz Robert T. (2011). Multi-voxel pattern analysis of fMRI data predicts clinical symptom severity. Neuroimage.

[bib0080] Craik F.I., Lockhart R.S. (1972). Levels of processing: a framework for memory research. J. Verbal Learn. Verbal Behav..

[bib0085] Danker J.F., Anderson J.R. (2010). The ghosts of brain states past: remembering reactivates the brain regions engaged during encoding. Psychol. Bull..

[bib0090] Daselaar S., Cabeza R., Cabeza R., Nyberg L., Park D.C. (2005). Age-related changes in hemispheric organization. Cognitive Neuroscience of Aging: Linking Cognitive and Cerebral Aging.

[bib9015] Detre G., Polyn S.M., Moore C., Natu V., Singer B., Cohen J., Haxby J.V., Norman K.A. (2006). The multi-voxel pattern analysis (MVPA) toolbox.

[bib0095] de Chastelaine M., Wang T.H., Minton B., Muftuler L.T., Rugg M.D. (2011). The effects of age, memory performance, and callosal integrity on the neural correlates of successful associative encoding. Cereb. Cortex.

[bib0100] Fujishiro H., Umegaki H., Suzuki Y., Oohara-Kurotani S., Yamaguchi Y., Iguchi A. (2005). Dopamine D-2 receptor plays a role in memory function: implications of dopamine-acetylcholine interaction in the ventral hippocampus. J. Psychopharmacol..

[bib0105] Gardiner J.M. (1988). Functional aspects of recollective experience. Mem. Cogn..

[bib0110] Goh J.O., Suzuki A., Park D.C. (2010). Reduced neural selectivity increases fMRI adaptation with age during face discrimination. NeuroImage.

[bib0115] Haxby J.V., Gobbini M.I., Furey M.L., Ishai A., Schouten J.L., Pietrini P. (2001). Distributed and overlapping representations of faces and objects in ventral temporal cortex. Science.

[bib9020] Haynes John-Dylan, Rees Geraint (2006). Decoding mental states from brain activity in humans. Nature Rev. Neurosci..

[bib9025] Head D., Rodrigue K.M., Kennedy K.M., Raz N. (2008). Neuroanatomical and cognitive mediators of age-related differences in episodic memory. Neuropsychology.

[bib0120] Hofer S.M., Sliwinski M.J. (2001). Understanding ageing — an evaluation of research designs for assessing the interdependence of ageing-related changes. Gerontology.

[bib0125] Honey G., Bullmore E. (2004). Human pharmacological MRI. Trends Pharmacol. Sci..

[bib0130] Johnson J.D., McDuff S.G.R., Rugg M.D., Norman K.A. (2009). Recollection, familiarity, and cortical reinstatement: a multivoxel pattern analysis. Neuron.

[bib0135] Kaasinen V., Vilkman H., Hietala J., Nagren K., Helenius H., Olsson H., Farde L., Rinne J.O. (2000). Age-related dopamine D2/D3 receptor loss in extrastriatal regions of the human brain. Neurobiol. Aging.

[bib9030] Kamitani Y., Tong F. (2005). Decoding the visual and subjective contents of the human brain. Nature Neurosci..

[bib0140] Kuhl B.A., Rissman J., Chun M.M., Wagner A.D. (2011). Fidelity of neural reactivation reveals competition between memories. Proc. Natl. Acad. Sci. U. S. A..

[bib0145] Levine B., Svoboda E., Hay J.F., Winocur G., Moscovitch M. (2002). Aging and autobiographical memory: dissociating episodic from semantic retrieval. Psychol. Aging.

[bib0150] Li S.C., Lindenberger U., Sikstrom S. (2001). Aging cognition: from neuromodulation to representation. Trends Cogn. Sci..

[bib0155] Li S.C., Naveh-Benjamin M., Lindenberger U. (2005). Aging neuromodulation impairs associative binding — a neurocomputational account. Psychol. Sci..

[bib0160] Li S.-C., Papenberg G., Nagel I.E., Preuschhof C., Schroeder J., Nietfeld W., Bertram L., Heekeren H.R., Lindenberger U., Baeckman L. (2013). Aging magnifies the effects of dopamine transporter and D2 receptor genes on backward serial memory. Neurobiol. Aging.

[bib0165] Luciana M., Collins P.F., Depue R.A. (1998). Opposing roles for dopamine and serotonin in the modulation of human spatial working memory functions. Cereb. Cortex.

[bib0170] Marr D. (1982). Vision.

[bib0175] Mattay V.S., Fera F., Tessitore A., Hariri A.R., Berman K.F., Das S., Meyer-Lindenberg A., Goldberg T.E., Callicott J.H., Weinberger D.R. (2006). Neurophysiological correlates of age-related changes in working memory capacity. Neurosci. Lett..

[bib0180] McClelland J.L., McNaughton B.L., Oreilly R.C. (1995). Why there are complementary learning-systems in the hippocampus and neocortex — insights from the successes and failures of connectionist models of learning and memory. Psychol. Rev..

[bib0185] McDuff S.G.R., Frankel H.C., Norman K.A. (2009). Multivoxel pattern analysis reveals increased memory targeting and reduced use of retrieved details during single-agenda source monitoring. J. Neurosci..

[bib0190] Mehta M.A., Hinton E.C., Montgomery A.J., Bantick R.A., Grasby P.M. (2005). Sulpiride and mnemonic function: effects of a dopamine D2 receptor antagonist on working memory, emotional memory and long-term memory in healthy volunteers. J. Psychopharmacol..

[bib0195] Mehta M.A., Montgomery A.J., Kitamura Y., Grasby P.M. (2008). Dopamine D2 receptor occupancy levels of acute sulpiride challenges that produce working memory and learning impairments in healthy volunteers. J. Psychopharmacol. (Berl).

[bib0205] Morcom A.M., Johnson W. (2015). Neural reorganization and compensation in aging. J. Cogn. Neurosci..

[bib0200] Morcom A.M., Bullmore E.T., Huppert F.A., Lennox B., Praseedom A., Linnington H., Fletcher P.C. (2010). Memory encoding and dopamine in the aging brain: a psychopharmacological neuroimaging study. Cereb. Cortex.

[bib9035] Morcom Alexa M. (2003). Age effects on the neural correlates of successful memory encoding. Brain.

[bib0210] Müller U., von Cramon D.Y., Pollmann S. (1998). D1-versus D2-receptor modulation of visuospatial working memory in humans. J. Neurosci..

[bib0215] Nelson H.E. (1982). The National Adult Reading Test (NART).

[bib9040] Norman K.A., Polyn S.M., Detre G.J., Haxby J.V. (2006). Beyond mind-reading: multi-voxel pattern analysis of fMRI data. Trends cognit. sci..

[bib0220] Oranje B., Gispen-de Wied C.C., Westenberg H.G.M., Kemner C., Verbaten M.N., Kahn R.S. (2004). Increasing dopaminergic activity: effects of L-dopa and bromocriptine on human sensory gating. J. Psychopharmacol..

[bib0225] O'Reilly R.C., McClelland J.L. (1994). Hippocampal conjunctive encoding, storage, and recall: avoiding a trade-off. Hippocampus.

[bib9045] Otmakhova Nonna A., Lisman John E. (1998). "D1/D5 dopamine receptors inhibit depotentiation at CA1 synapses via cAMP-dependent mechanism.". J. Neurosci..

[bib9050] Otten Leun J., Rugg Michael D. (2001). "Task-dependency of the neural correlates of episodic encoding as measured by fMRI.". Cereb. Cortex.

[bib0230] Papenberg G., Backman L., Nagel I.E., Nietfeld W., Schroeder J., Bertram L., Heekeren H.R., Lindenberger U., Li S.-C. (2013). Dopaminergic gene polymorphisms affect long-term forgetting in old age: further support for the magnification hypothesis. J. Cogn. Neurosci..

[bib0235] Papenberg G., Baeckman L., Nagel I.E., Nietfeld W., Schroeder J., Bertram L., Heekeren H.R., Lindenberger U., Li S.-C. (2014). COMT polymorphism and memory dedifferentiation in old age. Psychol. Aging.

[bib0240] Park D.C., Polk T.A., Park R., Minear M., Savage A., Smith M.R. (2004). Aging reduces neural specialization in ventral visual cortex. Proc. Natl. Acad. Sci. U. S. A..

[bib0245] Park J., Carp J., Hebrank A., Park D.C., Polk T.A. (2010). Neural specificity predicts fluid processing ability in older adults. J. Neurosci..

[bib0250] Park J., Carp J., Kennedy K.M., Rodrigue K.M., Bischof G.N., Huang C.-M., Rieck J.R., Polk T.A., Park D.C. (2012). Neural broadening or neural attenuation? Investigating age-related dedifferentiation in the face network in a large lifespan sample. J. Neurosci..

[bib0255] Payer D., Marshuetz C., Sutton B., Hebrank A., Welsh R.C., Park D.C. (2006). Decreased neural specialization in old adults on a working memory task. Neuroreport.

[bib0260] Polyn S.M., Natu V.S., Cohen J.D., Norman K.A. (2005). Category-specific cortical activity precedes retrieval during memory search. Science.

[bib9055] Poppenk J., Norman K.A. (2012). Mechanisms supporting superior source memory for familiar items: A multi-voxel pattern analysis study. Neuropsychologia.

[bib9060] Pralong E., Jones R.S.G. (1993). "Interactions of Dopamine with Glutamate‐and GABA‐mediated Synaptic Transmission in the Rat Entorhinal Cortex In Vitro.". European J. Neurosci..

[bib0265] Reddymasu S.C., Soykan I., McCallum R.W. (2007). Domperidone: review of pharmacology and clinical applications in gastroenterology. Am. J. Gastroenterol..

[bib0270] Reeves S., Mehta M., Howard R., Grasby P., Brown R. (2010). The dopaminergic basis of cognitive and motor performance in Alzheimer's disease. Neurobiol. Dis..

[bib0275] Ritchey M., Wing E.A., Labar K.S., Cabeza R. (2013). Neural similarity between encoding and retrieval is related to memory via hippocampal interactions.". Cerebral Cortex.

[bib0280] Schacter D.L., Koutstaal W., Norman K.A. (1997). False memories and aging. Trends Cogn. Sci..

[bib0285] Snodgrass J.G., Corwin J. (1988). Pragmatics of measuring recognition memory: applications to dementia and amnesia. J. Exp. Psychol..

[bib0290] Spencer W.D., Raz N. (1995). Differential effects of aging on memory for content and context: a meta-analysis. Psychol. Aging.

[bib0300] Staresina B.P., Henson R.N.A., Kriegeskorte N., Alink A. (2012). Episodic reinstatement in the medial temporal lobe. J. Neurosci..

[bib0305] Stemmelin J., Lazarus C., Cassel S., Kelche C., Cassel J.C. (2000). Immunohistochemical and neurochemical correlates of learning deficits in aged rats. Neuroscience.

[bib0295] St-Laurent M., Abdi H., Bondad A., Buchsbaum B.R. (2014). Memory reactivation in healthy aging: evidence of stimulus-specific dedifferentiation. J. Neurosci..

[bib0310] Talairach J., Tournoux P. (1988). Co-planar Stereotaxic Atlas of the Human Brain.

[bib0315] Treves A., Rolls E.T. (1994). Computational analysis of the role of the hippocampus in memory. Hippocampus.

[bib0320] Umegaki H., Munoz J., Meyer R.C., Spangler E.L., Yoshimura J., Ikari H., Iguchi A., Ingram D.K. (2001). Involvement of dopamine D-2 receptors in complex maze learning and acetylcholine release in ventral hippocampus of rats. Neuroscience.

[bib0325] Von Bahr C., Wiesel F.A., Movin G., Eneroth P., Jansson P., Nilsson L., Ogenstad S. (1991). Neuroendocrine responses to single oral doses of remoxipride and sulpiride in healthy female and male volunteers. Psychopharmacology.

[bib0330] Wagner A.D., Davachi L. (2001). Cognitive neuroscience: forgetting of things past. Curr. Biol..

[bib0335] West R.L. (1996). An application of prefrontal cortex function theory to cognitive aging. Psychol. Bull..

[bib0340] Wiesel F.A., Alfredsson G., Ehrnebo M., Sedvall G. (1982). Prolactin response following intravenous and oral sulpiride in healthy human subjects in relation to sulpiride concentrations. Psychopharmacology.

[bib0345] Wilson I.A., Gallagher M., Eichenbaum H., Tanila H. (2006). Neurocognitive aging: prior memories hinder new hippocampal encoding. Trends Neurosci..

[bib9065] Yassa M.A., Lacy J.W., Stark S.M., Albert M.S., Gallagher M., Stark C.E. (2011). Pattern separation deficits associated with increased hippocampal CA3 and dentate gyrus activity in nondemented older adults. Hippocampus.

[bib9070] Yonelinas A.P., Widaman K., Mungas D., Reed B., Weiner M.W., Chui H.C. (2007). Memory in the aging brain: doubly dissociating the contribution of the hippocampus and entorhinal cortex. Hippocampus.

[bib9075] Zhang J., Yang Y. (2003). Robustness of Regularized Linear Classification Methods in Text Categorization. Proceedings of SIGIR-2003. 26st ACM International Conference on Research and Development in Information Retrieval.

