# Supplementary material for: Dopamine and memory dedifferentiation in aging
Source: Neuroimage. 2017 Jun;153:211–20. doi: 10.1016/j.neuroimage.2015.03.031 (PMC5460975; doi:10.1016/j.neuroimage.2015.03.031)
Supplement: Inline Supplementary Table S1 [file mmc1.docx]

Table S1. Task specificity (study phase) under Placebo (ridge regression). The mean (SD) accuracy of discrimination between semantic and phonological task blocks in the study (encoding) phase of the task is listed (see Materials and Methods for details of analyses and Results for statistical analysis).

| ROI/# voxels | Younger group | | | Older group | | | |
| --- | --- | --- | --- | --- | --- | --- | --- |
|  | 50 | 150 | 500 | | 50 | 150 | 500 |
| LIFG (500) | 0.82 (0.07) | 0.83 (0.07) | 0.84 (0.07) | | 0.80 (0.07) | 0.81 (0.07) | 0.82 (0.07) |
| RIFG (500) | 0.80 (0.07) | 0.81 (0.08) | 0.80 (0.09) | | 0.76 (0.06) | 0.77 (0.07) | 0.77 (0.08) |
| LMFG (500) | 0.80(0.07) | 0.82 (0.06) | 0.83 (0.06) | | 0.80 (0.06) | 0.82 (0.05) | 0.83 (0.05) |
| RMFG (500) | 0.80 (0.06) | 0.83 (0.06) | 0.84 (0.06) | | 0.80 (0.07) | 0.81 (0.07) | 0.82 (0.07) |
| HC (50) | 0.69 (0.04) | 0.69 (0.05) | 0.63 (0.09) | | 0.72 (0.07) | 0.72 (0.07) | 0.70 (0.09) |
| LSOG (150) | 0.73 (0.07) | 0.73 (0.08) | 0.71 (0.08) | | 0.76 (0.08) | 0.77 (0.09) | 0.77 (0.09) |
| FusG (150) | 0.76 (0.06) | 0.78 (0.06) | 0.78 (0.07) | | 0.76 (0.06) | 0.78 (0.06) | 0.77 (0.07) |
