# Supplementary material for: Dopamine and memory dedifferentiation in aging
Source: Neuroimage. 2017 Jun;153:211–20. doi: 10.1016/j.neuroimage.2015.03.031 (PMC5460975; doi:10.1016/j.neuroimage.2015.03.031)
Supplement: Inline Supplementary Table S2 [file mmc2.docx]

Table S2. Drug effects on memory specificity (ridge regression). Means (SDs) are given for analyses of the selected feature sets in the Sulpiride, Placebo and Bromocriptine conditions (see Table 1 for details).

| ROI (# voxels)/ Drug session | Younger group | | | Older group | | |
| --- | --- | --- | --- | --- | --- | --- |
|  | Sulpiride | Placebo | Bromocriptine | Sulpiride | Placebo | Bromocriptine |
| LIFG (500) | 0.14 (0.11) | 0.23 (0.18) | 0.16 (0.14) | 0.11 (0.14) | 0.05 (0.15) | 0.13 (0.13) |
| RIFG (500) | 0.08 (0.14) | 0.14 (0.12) | 0.07 (0.12) | 0.09 (0.15) | 0.09 (0.18) | 0.03 (0.14) |
| LMFG (500) | 0.14 (0.08) | 0.18 (0.14) | 0.16 (0.13) | 0.08 (0.14) | 0.06 (0.13) | 0.08 (0.11) |
| RMFG (500) | 0.08(0.09) | 0.16 (0.12) | 0.07 (0.17) | 0.12 (0.11) | 0.10 (0.16) | 0.11 (0.14) |
| HC (50) | 0.01 (0.12) | 0.12 (0.14) | 0.06 (0.10) | 0.05 (0.15) | 0.01 (0.10) | 0.05 (0.13) |
| LSOG (150) | 0.07 (0.11) | 0.12 (0.11) | 0.15 (0.15) | 0.08 (0.15) | 0.11 (0.09) | 0.06 (0.18) |
| FusG (150) | 0.08 (0.15) | 0.11 (0.18) | 0.02 (0.12) | 0.05 (0.15) | 0.02 (0.14) | 0.05 (0.17) |
