# Supplementary material for: Dopamine and memory dedifferentiation in aging
Source: Neuroimage. 2017 Jun;153:211–20. doi: 10.1016/j.neuroimage.2015.03.031 (PMC5460975; doi:10.1016/j.neuroimage.2015.03.031)
Supplement: Inline Supplementary Table S3 [file mmc3.docx]

Table S3. Drug effects on memory specificity (correlation distance metric). Means (SDs) are given for the selected feature sets for the difference: within-task correlation - between task correlation. Both correlation measures are among semantic and phonological task blocks across the study (encoding) phase and the test (retrieval) phase of the task (see Materials and Methods for details of analyses and Results for statistical analysis).

| ROI (# voxels)/ Drug session | Younger group | | | Older group | | |
| --- | --- | --- | --- | --- | --- | --- |
|  | Sulpiride | Placebo | Bromocriptine | Sulpiride | Placebo | Bromocriptine |
| LIFG | 0.22 (0.38) | 0.41 (0.27) | 0.40 (0.35) | 0.04 (0.45) | 0.02 (0.28) | 0.08 (0.45) |
| RIFG | 0.09 (0.38) | 0.21 (0.28) | 0.09 (0.33) | 0.14 (0.42) | -0.02 (0.43) | 0.00 (0.38) |
| LMFG | 0.21 (0.37) | 0.38 (0.34) | 0.41 (0.44) | 0.03 (0.59) | 0.03 (0.34) | 0.09 (0.48) |
| RMFG | 0.08(0.35) | 0.29 (0.27) | 0.15 (0.53) | 0.14 (0.50) | 0.06 (0.44) | 0.12 (0.41) |
| HC | 0.10 (0.36) | 0.28 (0.47) | 0.15 (0.48) | 0.11 (0.53) | 0.06 (0.37) | -0.07 (0.64) |
| LSOG | 0.16 (0.33) | 0.42 (0.30) | 0.20 (0.66) | 0.18 (0.52) | 0.25 (0.37) | 0.11 (0.66) |
| FusG | 0.15 (0.43) | 0.28 (0.42) | 0.11 (0.41) | 0.09 (0.46) | -0.01 (0.41) | -0.02 (0.52) |
