# Supplementary material for: Dopamine and memory dedifferentiation in aging
Source: Neuroimage. 2017 Jun;153:211–20. doi: 10.1016/j.neuroimage.2015.03.031 (PMC5460975; doi:10.1016/j.neuroimage.2015.03.031)
Supplement: Supplementary file 4 — Supplementary material. [file mmc4.docx]

**Supplementary Information**

*Session ordering matched analyses*

Due to the exclusion of some subjects, there were minor differences in the ordering of drug sessions between the two age groups, although these were not significant (across the 6 balanced drug orderings, *χ^2^*(5) = .58, n.s.). To check that the findings of dopaminergic modulation were not artifacts of these minor differences, we performed several additional analyses. In each case these balanced session order for the critical effect, and maximised N.

Placebo condition: age x *Pr* effects on Memory Specificity

In the main analysis of group differences, the session on which the Placebo had been received was perfectly matched between the two age groups (N=16,16). However for the ANCOVA including *Pr* (N=16,15), there was one fewer older than young subject who had received Placebo on Session 3. For both ridge and correlation measures, the Group x *Pr* interaction was robust (*F*(1,26) = 7.60, p = .011; *F*(1,26) = 9.95, p = .004, respectively) and also remained reliable with age in the model (p = .013 and .008).

Dopaminergic effects: drug main effects on Task Specificity

To check that the substantial main effects of Drug on Task Specificity in all ROIs were not artifacts of session order, the check analysis included 2 subjects in each groups for each of the 6 drug session orders (N=12,12). These effects remained significant (p < .001 for all).

Dopaminergic effects: drug by performance by age effect on Memory Specificity

For the interaction of Group x Drug x *Pr* in Hippocampus, matching for drug session order yielded sample sizes of 13 and 13 (including exclusion of the subject with an outlier *Pr* value). This 3-way interaction for Memory Specificity was robust for both ridge and correlation analyses (*F*(1.8,39.6) = 7.97, p = .002; *F*(1.9,41.6) = 6.32, p = .005), and also significant in both cases with age in the model (p = .001and p = .010).
